# Identification of Adamts4 as a novel adult cardiac injury biomarker with therapeutic implications in patients with cardiac injuries

**DOI:** 10.1038/s41598-022-13918-3

**Published:** 2022-06-14

**Authors:** Riffat Khanam, Arunima Sengupta, Dipankar Mukhopadhyay, Santanu Chakraborty

**Affiliations:** 1 Department of Life Sciences, Presidency University, Kolkata, 700073 India; 2 Department of Life Science and Biotechnology, Jadavpur University, Kolkata, 700032 India; 3 Department of Cardiology, Institute of Post Graduate Medical Education and Research (IPGME&R), SSKM Hospital, Kolkata, 700020 India

**Keywords:** Cell biology, Developmental biology, Biomarkers, Cardiology

## Abstract

Pathological cardiac remodeling as an aftermath of a severe cardiac injury can lead to ventricular dysfunction and subsequent heart failure. Adamts4, a metalloproteinase, and disintegrin with thrombospondin-like motif, involved in the turnover of certain extracellular matrix molecules and pathogenesis of osteoarthritis, also plays a role in cardiac remodeling although little is presently known about its expression and function in the heart. Here, we have investigated the dynamic expression pattern of Adamts4 during cardiogenesis and also in the adult heart. To our surprise, adult cardiac injury reactivated Adamts4 expression concomitant with fibrosis induction. To better understand the mechanism, cultured H9c2 cardiomyocyte cells were subjected to ROS injury and Hypoxia. Moreover, through combinatorial treatment with SB431542 (an inhibitor of Tgf-β1), and Adamts4 siRNA mediated gene knockdown, we were able to decipher a regulatory hierarchy to the signal cascade being at the heart of Tgf-β regulation. Besides the hallmark expression of Adamts4 and Tgf-β1, expression of other fibrosis-related markers like Collagen-III, alpha-SMA and Periostin were also assessed. Finally, increased levels of Adamts4 and alpha-SMA proteins in cardiac patients also resonated well with our animal and cell culture studies. Overall, in this study, we highlight, Adamts4 as a novel biomarker of adult cardiac injury.

## Introduction

Heart failure remains a global cause of mortality and morbidity and an issue of public health concern with over 20 million people diagnosed with at least first-time heart failure across the world^1,2^. By 2016, at least 30 and 50 million cases of cardiovascular diseases (CVD) were recorded in the US and India respectively with a mortality rate of more than 70% in India^3^. Extracellular matrix (ECM) remodeling in heart is one of the contributing factors of pathogenesis in cardiovascular diseases^4,5^. Collagen-I forms the major component of matrix interstitium of the myocardium, the other components of ECM include Collagen-III, fibronectin, proteoglycans, matrix metalloproteinases (MMPs), and tissue inhibitors of matrix metalloproteinases (TIMPs). The proportion and biochemistry of the ECM change due to underlying pressure overload, cardiac injury, myocardial infarction (MI), and/or ischemia–reperfusion (I/R) injury that leads to extracellular matrix reorganization which in turn is modulated by changes in turnover of matrix proteins^6^. A typical cardiac remodeling post-cardiac injury undergoes, three major phases namely inflammatory, proliferative and maturation leading to a mature scar formation^7^.

Although the initial stages of ECM remodeling are essential as it prevents rupture of the ventricular wall and prevents ventricular dilatation, however, extensive and unregulated ECM remodeling leads to progressive fibrosis in the heart and impairment of cardiac functioning^8–10^.

The MMPs are a family of zinc-dependent proteases involved in the turnover of collagen^11^. An important MMP is the Adamts family which apart from inhabiting the functions of an MMP also acts as disintegrins. Adamts4 a member of the Adamts family is a metalloproteinase and a disintegrin with thrombospondin like motifs^10,12^. Adamts4 has been known to bind to the ECM proteins and executes cleavage of ECM proteoglycans like aggrecan, versican and brevican apart from regulating collagen turnover. There is not sufficient information in the context of cardiac remodeling with a focus on the involvement of Adamts4. So far it is known that Adamts4 knockout in animal models leads to a reduction in plaques in cases of high fat induced atherosclerosis^13^ and also, inhibition of Adamts4 with pentosan polysulfate following aortic banding improves cardiac functioning^6^. However, there is not much literature about Adamts4 expression and function in the developing and adult heart at the basal level and also in post cardiac injury. Only recently studies have shown that Adamts4 along with Adamts1 levels remain elevated in patients with acute aortic dissection and also in patients with coronary artery disease^14,15^.

Here in our present study, for the first time, we aim to identify Adamts4 as a novel biomarker of adult cardiac injury under stress conditions. We have detected a strong expression of Adamts4 protein in the developing cardiac chamber myocardium in utero compared to very restricted expression in the adult murine hearts. To our surprise, we have also detected the expression of Adamts4 in vivo murine model of myocardial infarction (MI), localized in adult cardiomyocytes. To better understand the molecular insights of Adamts4 induction and associated affected signaling pathway activation, we have used H9c2, a rat ventricular myoblast cell line for several in vitro assays. Likewise, Adamts4 expression was induced in H9c2 cells, subjected to hypoxia (Hyp) and ROS injury inductions in vitro. Moreover, we manipulated the expression of Adamts4 with siRNA-mediated loss of function and TGF-β inhibitor studies in H9c2 cells to evaluate its regulation and dependency on TGF-β signaling since TGF-β has been long known to be a characteristic marker for inflammatory and fibrotic responses following pathological stress including MI, ischemia and reperfusion (I/R) injury^16–20^. Finally and most importantly, we also validated our hypothesis in human clinical samples and demonstrated the induced expression of ADAMTS4 in patients with indicated cardiac ailments.

## Results

### Dynamic expression pattern of murine Adamts4 protein observed in developing and adult hearts

**Figure 1:**
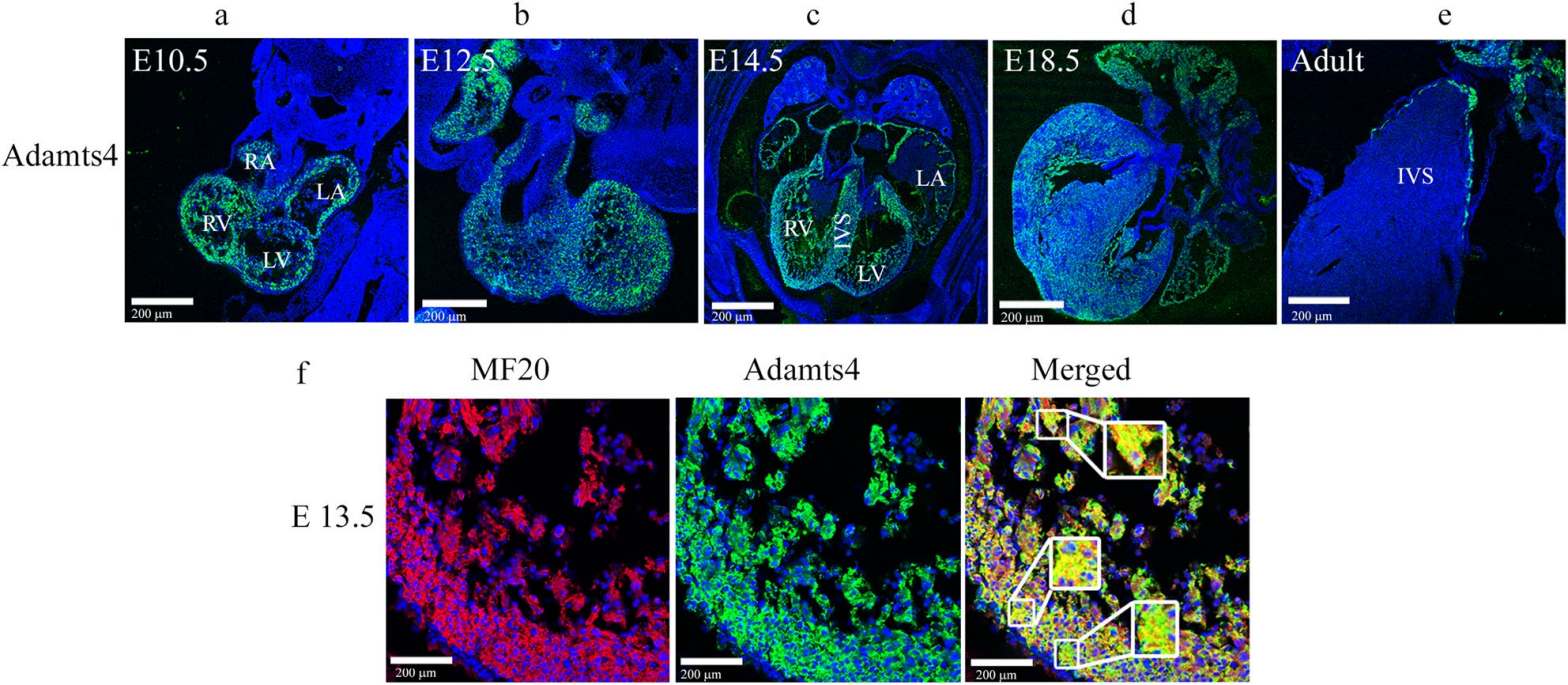
Dynamic expression of Adamts4 protein in embryonic and adult heart. Adamts4 expression is shown in developing E10.5 (**a**), E12.5 (**b**), E14.5 (**c**), E18.5 (**d**) adult (**e**) murine hearts. IHC with anti-Adamts4 antibody (green colour) and Topro3 (blue colour) used as nuclear stain, the expression pattern of Adamts4 is observed in (**a**)–(**e**). While the expression pattern of Adamts4 is more widespread in the embryonic stages throughout the RA, LA, RV, LV and IVS (**a**–**d**), the expression drastically reduces and is mainly only confined at the IVS in the adult stage (**e**) displaying a sharp contrast between the expression of Adamts4 in developing and adult stages. (**f**) Highlights the colocalization of Adamts4 (green) in the chamber myocardium with E13.5 chamber cardiomyocytes shows colocalization of Adamts4 with MF20 (shown in yellow colour) across the LV of chamber myocardium. The arrowheads point towards cells that have colocalized with both the markers ie, Adamts4 and MF20. This confirms the expression of Adamts4 in embryonic cardiomyocytes. n = 4.

Immunohistochemistry (IHC) with anti-Adamts4 antibody shows Adamts4 protein expresses strongly in developing murine cardiac chambers but the expression significantly wanes in adult murine hearts. In contrast to the adult heart (Fig. 1e) where Adamts4 expression is largely restricted at the edge of the interventricular septum (IVS) mostly adjacent to the left ventricle (LV), in the embryonic heart (Fig. 1a–d) the expression of Adamts4 is more widespread throughout the left and right auricles (RA, LA), ventricles (LV and RV) and the inter-ventricular septum (IVS) of the chamber myocardium of E10.5, E12.5, E14.5, and E18.5 although its expression in endothelial-derived heart valves remained mostly inconspicuous. Further, the expression of Adamts4 at E13.5 (Fig. 1f) is also co-localized with cardiomyocyte-specific marker MF20 (MF20 is a myosin heavy chain-II marker in cardiac and skeletal muscle system, hence often used to label sections of myocardium and is therefore considered to be cardiomyocyte marker as well^21,22^.) throughout the LV of chamber myocardium.

### Adamts4 reactivation in the ventricular chamber in the adult heart following injury in-vivo

**Figure 2:**
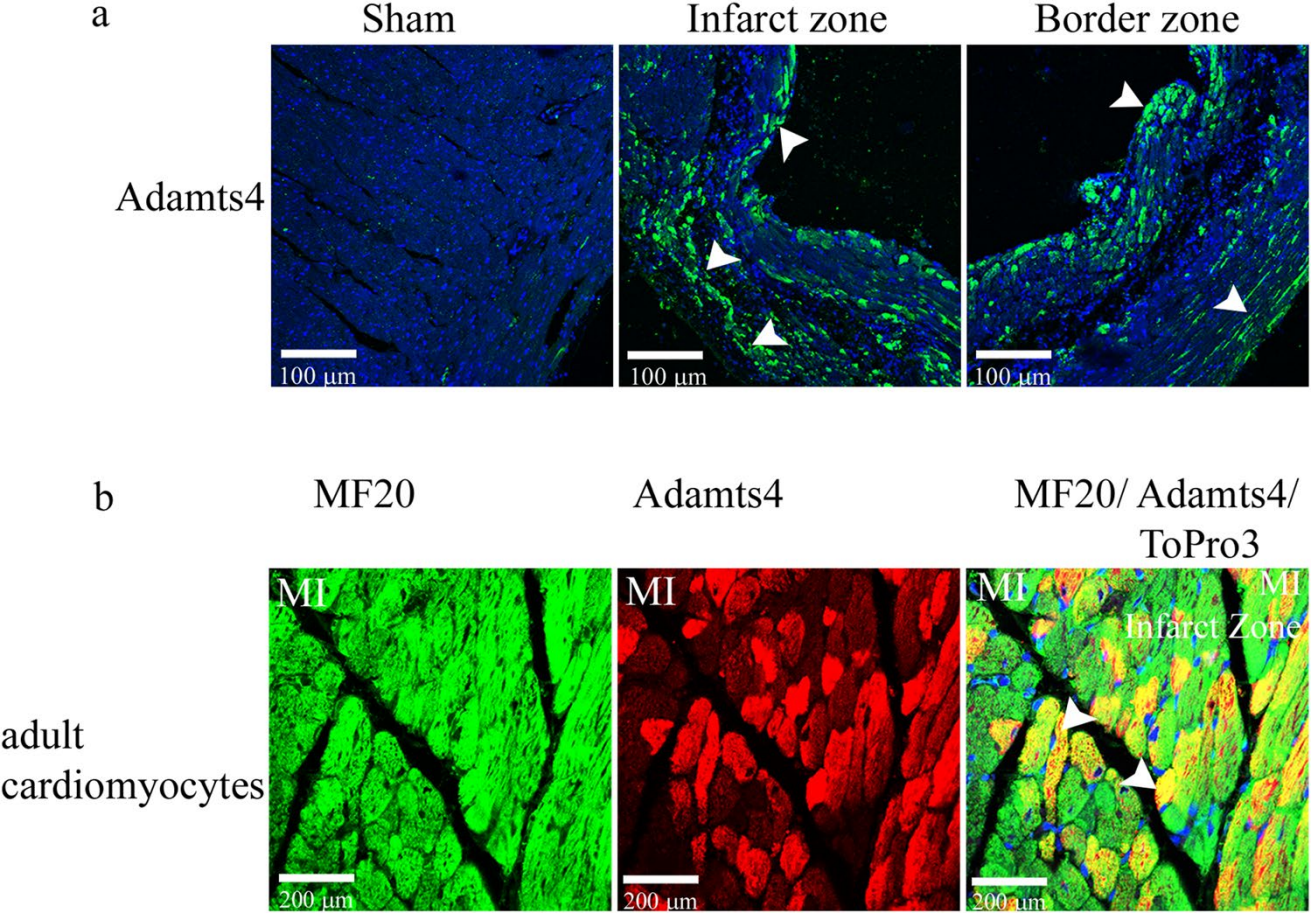
Adamts4 protein is reactivated in adult murine heart following 4 weeks post-MI. Panel a. shows IHC with anti-Adamts4 antibody (shown in green colour) and Topro3, used as nuclear stain (shown in blue). 4 weeks post-MI, induced reactivation of murine Adamts4, as indicated by the arrowheads in the infarct and border zones of adult murine chamber myocardium in comparison to sham operated mice. Moreover, this injury induced expression of Adamts4 is expressed in cardiomyocytes as shown in panel b where IHC with anti-Adamts4 (shown in red colour) and cardiomyocyte specific anti-MF20 (shown in green colour) antibodies following 4 weeks post MI in adult murine show colocalized expression, the colocalization is pointed out by the arrowheads in the MF20/Adamts4/Topro3 merged image. n = 4.

While Adamts4 expression in adult murine hearts significantly waned in comparison to developing hearts, it is found that MI-induced adult murine heart shows significant reactivation of Adamts4 protein. IHC with anti-Adamts4 antibody shows a strong expression of Adamts4 in the infarct and border zone (Fig. 2a) following 4 weeks post-MI in comparison to sham-operated mice. Again, this reactivated expression of Adamts4 following MI in adult mice is co-localized with MF20 as denoted by IHC with Adamts4 and MF20 (Fig. 2b) emphasizing that Adamts4 expression co-localizes with cardiomyocytes in adult hearts.

### Adamts4, Tgf-β1 upregulation in H9c2 cells following injury induction

**Figure 3:**
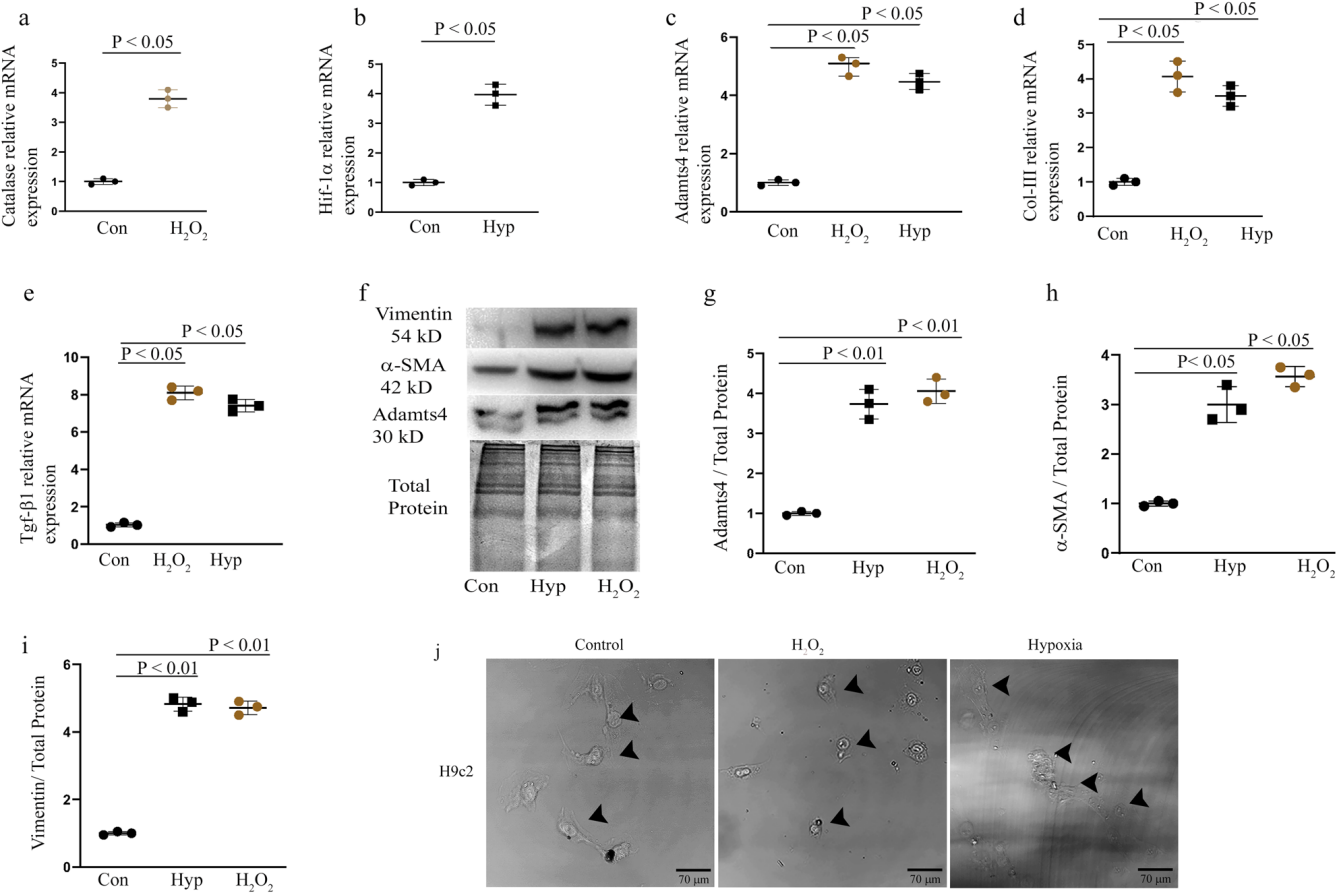
Injury induced overexpression of Adamts4 and fibrosis related markers in H9c2 cells. Relative mRNA expression assessed by quantitative real-time PCRs of Catalase (**a**) shows an upregulation by 3.8 fold, Hif-1α (**b**) was found to be elevated by fourfold, Adamts4 (**c**) was found to be upregulated by 5 and 4.5 fold for H_2_O_2_ and Hyp treatment sets respectively, Col-III (**d**) was found to be elevated by 4 and 3.5 fold H_2_O_2_ and Hyp treatments, Tgf-β1 (**e**) levels were upregulated by 8 and 7.4 folds for H_2_O_2_ and Hyp treatments respectively. Also, elevated expression of proteins analyzed by WB with Adamts4 (**f** and **g**) showed an enhanced expression of 3.7 and fourfold for Hyp and H_2_O_2_ treatments, α-SMA (**f** and **h**) showed an upregulation of 3 and 3.5 folds Hyp and H_2_O_2_ treatment groups, Vimentin (**f** and **i**) under stress induced conditions of hypoxia and H_2_O_2_ showed upregulated expression by 4.8 fold for hypoxia and 4.7 fold for H_2_O_2_ treatments. Elevated expression of markers-Catalase and Hif-1α signified successful injury inductions while upregulated expressions of Adamts4, Col-III, Tgf-β1, α-SMA and Vimentin indicated development of injury related fibrosis. ß-actin was used to normalize gene expression for qPCR assay and total protein was used as loading control for WB. n = 3. Data analyzed and expressed as mean ± SD. Differences were considered statistically significant for p < 0.05. (**j**) H9c2 morphology after injury induction as compared to control taken with the help of a DIC microscope.

To better understand the detailed mechanistic insights into Adamts4 function in post injury; further studies were done in H9c2, a rat cardiomyocyte cell line. H9c2 cells were subjected to H_2_O_2_ and Hypoxia (Hyp) treatment. Figure 3a, b show upregulation of ROS and hypoxia injury-related markers Catalase^23–25^ and Hif-1α^26^ respectively validated by real-time qPCR, showing upregulation after injury for both the markers. Also, Adamts4 and Collagen-III^27,28^ expression at mRNA levels shows elevation after both stress treatments (Fig. 3c, d). Furthermore, Tgf-β1 mRNA shows upregulation following H_2_O_2_ and Hypoxia treatment^18,29^ as quantified by real-time PCR (Fig. 3e). Moreover, Adamts4, α-SMA and Vimentin (a type-III intermediate filament marker that is known to induce repair mesenchymal cells following severe stress or injury to cells to initiate wound healing and provide structural support network^30,31^) expression at protein levels assessed by western blot (WB) shows significant upregulation following H_2_O_2_ and Hypoxia treatments (Fig. 3f–i). Figure 3j depicts H9c2 morphology after injury induction as compared to control taken in DIC mode and cell size measurements post injury induction is shown in Supplementary Fig. S1. Overall, this figure shows the upregulation of ECM markers like Col-III, α-SMA, Vimentin along with Adamts4 and Tgf-β1 following H_2_O_2_ and Hypoxia treatment. All qPCRs were normalized with β-actin and total protein was used as loading control for western blot assays.

### Adamts4 and Tgf-β1 expression is suppressed by SB431542 (ALKI) pre-treatment before H_2_O_2_ and Hypoxia stress induction

**Figure 4:**
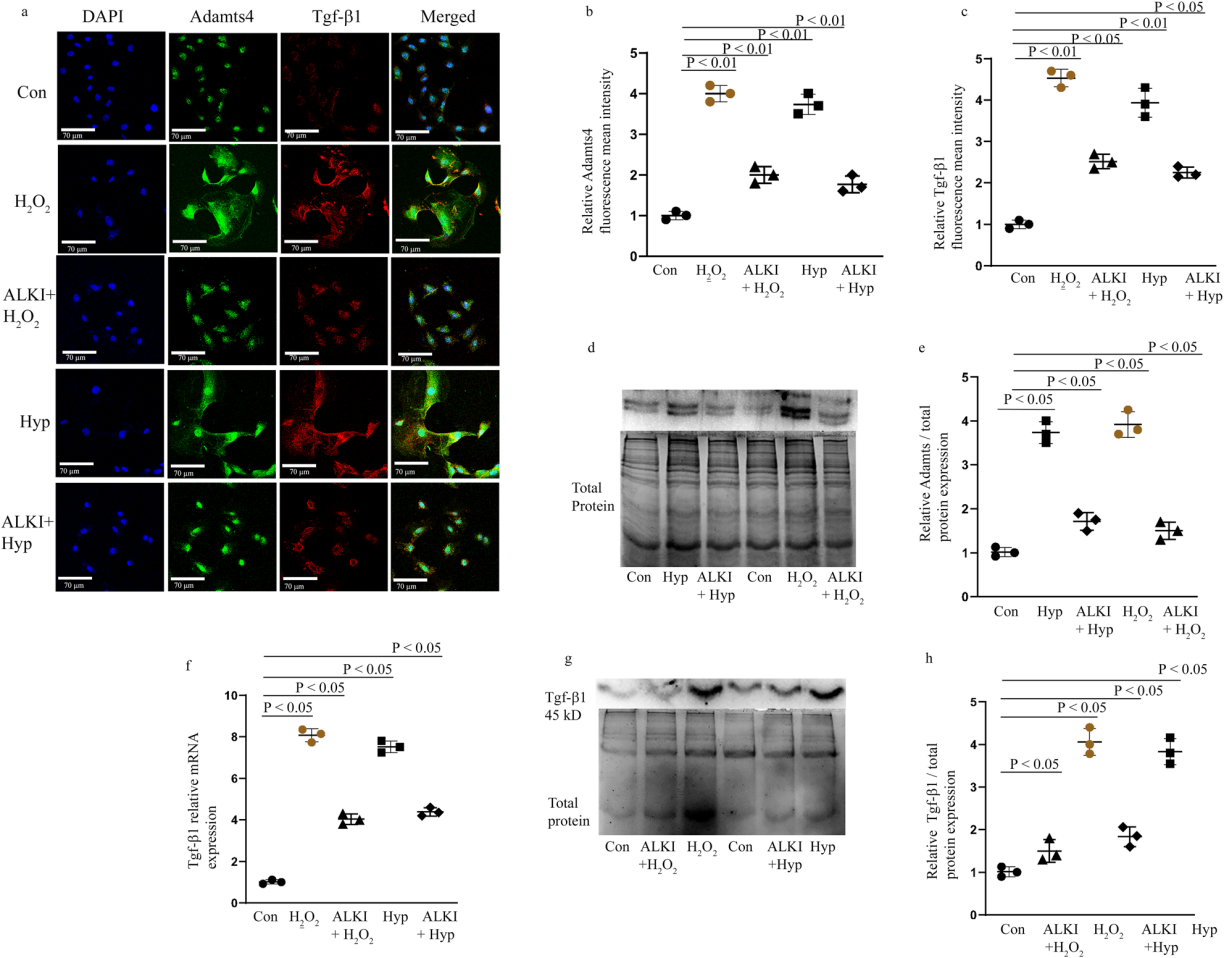
Adamts4 and Tgf-β1 expression is inhibited following pre-treatment with ALKI. Staining with anti-Adamts4 (shown in green) showed a 4- and 3.7-fold increase following H_2_O_2_ and hypoxia treatments in the fluorescence intensities of Adamts4 but was found to reduce to 2 and 1.6 folds in the treatment groups—ALKI + H_2_O_2_ and ALKI + Hypoxia respectively (**a** and **b**). IF with anti-Tgf-β1 antibody (shown in red) showed elevated expression of 4.5 and fourfold following H_2_O_2_ and hypoxia treatments and this reduced to 2.5 and 2.3 folds for ALKI + H_2_O_2_ and ALKI + Hypoxia groups (**a** and **c**). DAPI (in blue) was used as nuclear stain. Adamts4 WB shows of increased expression of 3.7 and 4 folds for the Hyp, H_2_O_2_ treated groups which reduce to 1.7, and 1.5 in ALKI + Hyp and ALKI + H_2_O_2_ treated groups (**d** and **e**). Tgf-β1 inhibition following ALKI pre-treatment was also assessed by qPCR (**f**) which showed a reduction from 8 and 7.4 folds for H_2_O_2_ and hypoxia treatment groups to 4 and 4.4 folds for ALKI + H_2_O_2_ and ALKI + Hyp treatment groups. Tgf-β1 protein expression measured by WB (**g** and **h**) showed a 4- and 3.8-fold increased change for H_2_O_2_ and Hypoxia subjected H9c2 cells to 1.5 and 1.8 folds for ALKI + H_2_O_2_ and ALKI + Hyp treatment respectively. β-actin was used to normalize gene expression for qPCR assay and total protein was used as loading control for WB. n = 3, data analyzed and expressed as mean ± SD. Differences were considered statistically significant for p < 0.05.

Immunofluorescence (IF) staining with anti-Adamts4 and anti-Tgf-β1 antibody shows an elevated expression of both the markers following H_2_O_2_ and Hypoxia treatment assessed by quantifying fluorescence mean intensity (via ImageJ software, NIH). Interestingly, this elevated expression significantly reduced for both Adamts4 and Tgf-β1 in presence of ALKI pre-treatment. Interesting to note, a non-specific nuclear presence of Adamts4 is observed irrespective of treatment (Fig. 4a–c) Further, the WB also shows a similar trend in the expression pattern of Adamts4 and Tgf-β1 proteins where the expression of both markers falls after ALKI pre-treatment in comparison to only injury groups (Fig. 4 d, e, g and h). Also, Tgf-β1 measured at mRNA levels by qPCR also validates the same finding (Fig. 4f). Overall, ALKI shows successful significant inhibition of Tgf-β1 as also stated previously^20,32,33^ along with inhibition of Adamts4. This finding is indicative of Tgf-β1 dependent activation of Adamts4.

### ALKI pre-treatment before H_2_O_2_ and Hypoxia treatment suppresses the expression of Collagen-III and α-SMA

**Figure 5:**
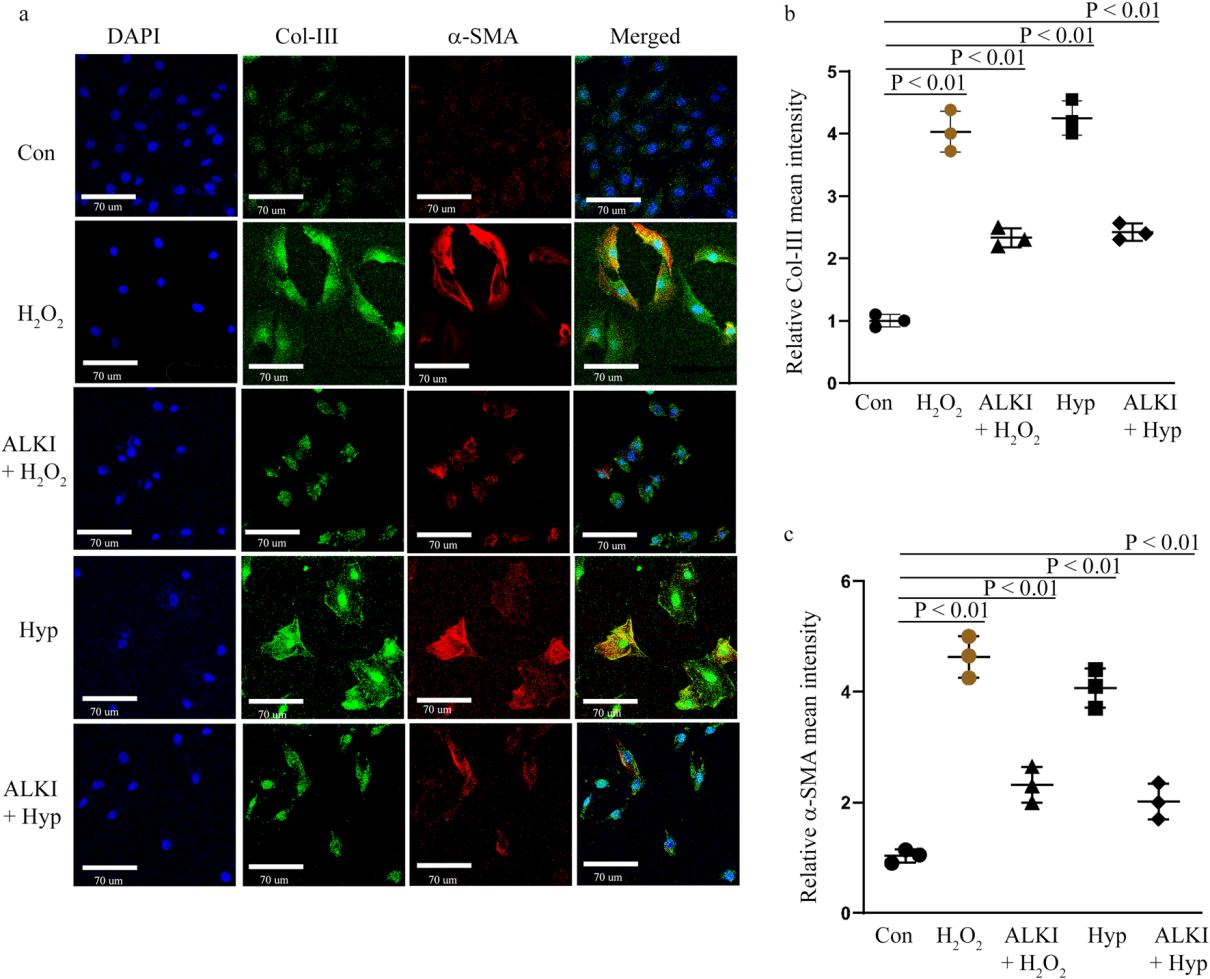
Inhibition of Col-III and α-SMA by ALKI pre-treatment. IF staining with anti-Col-III (shown in green) and anti-α-SMA (shown on red) antibodies showed inhibited expression of the markers under conditions of ALKI pre-treatment paired with H_2_O_2_ and hypoxia when compared to only injury states-H_2_O_2_ and hypoxia. Col-III expression reduced to 2.3 and 2.4 folds for ALKI + H_2_O_2_ and ALKI + Hypoxia treatment sets from 4 and 4.25 folds for H_2_O_2_ and Hyp treatments respectively (**a** and **b**). α-SMA levels were reduced to 2.3 and 2 folds for ALKI + H_2_O_2_ and ALKI + Hypoxia treatment groups from 4.6 and 4 folds for H_2_O_2_ and Hyp treatments respectively. DAPI (shown in blue was used as nuclear stain) n = 3, data analyzed and expressed as mean ± SD. Differences were considered statistically significant for p < 0.05.

Fibrosis markers Collagen-III (Col-III) and α-SMA^27,34,35^ IF staining show a reduction in expression of both markers in H_2_O_2_ and Hyp groups pre-treated with ALKI after a significant upregulation in the expression of both markers observed in the injury groups; H_2_O_2_ and Hyp as measured by assessing fluorescence intensities of Col-III and α-SMA (Fig. 5a–c). Overall, this suggests that the expression of Collagen-III and α-SMA may be Adamts4 and Tgf-β1 dependent.

### ALKI pre-treatment prior to H_2_O_2_ and Hypoxia treatment inhibits Periostin expression

**Figure 6:**
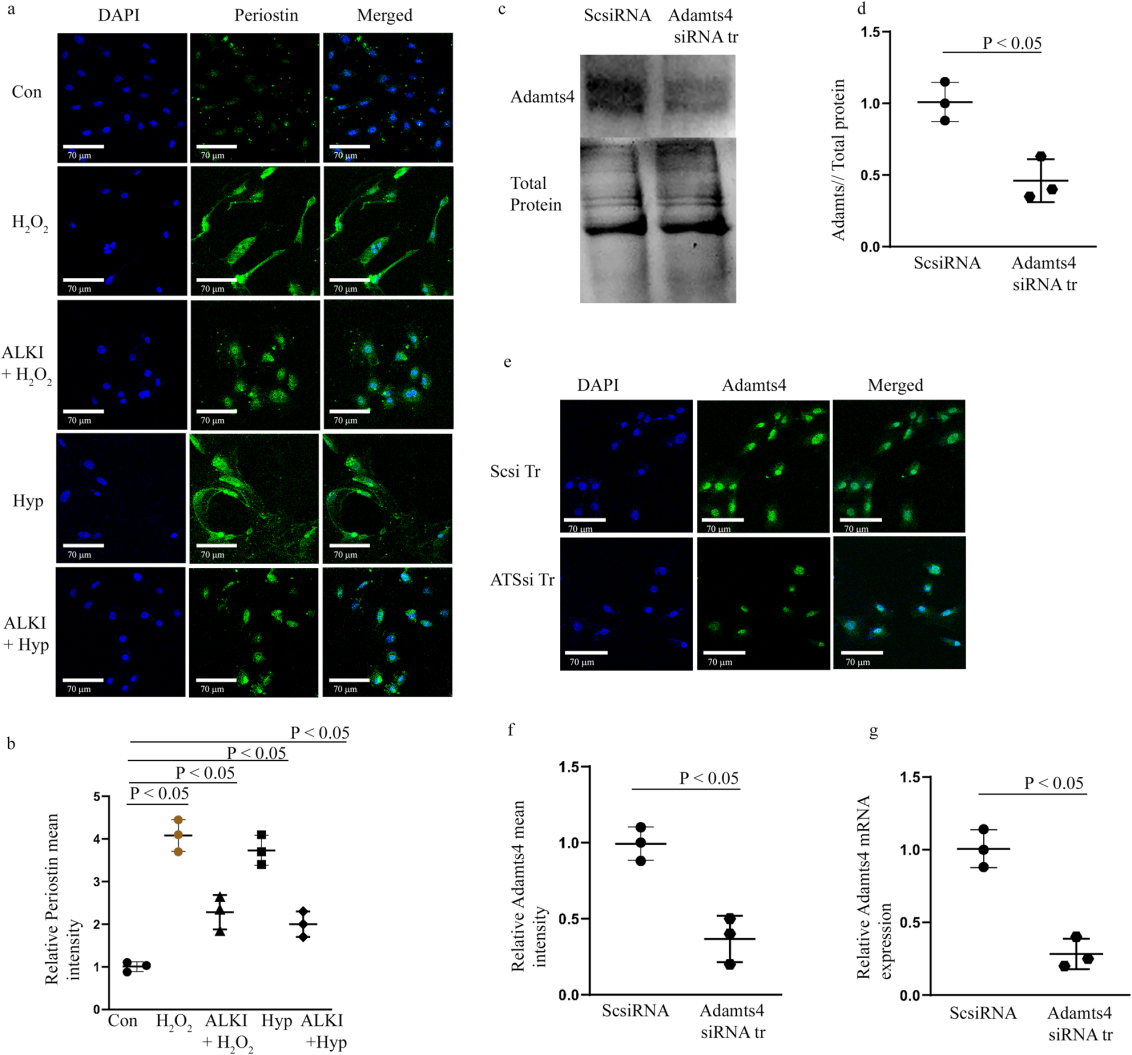
Periostin expression is reduced after ALKI pre-treatment and successful knockdown of Adamt4 mRNA in cultured H9c2 cells. IF with anti-Periostin antibody (shown in green) shows inhibition of Periostin expression under ALKI + H_2_O_2_, and ALKI + Hyp conditions as compared to only H_2_O_2_ and Hyp treated conditions. A reduced expression of 2.3 and 2 folds for ALKI + H_2_O_2_, and ALKI + Hyp was observed which was a decrease from the 4 and 3.7 fold change found for only H_2_O_2_ and Hyp treatments (**a** and **b**) in comparison to control. Successful knockdown of Adamts4 via Adamts4 siRNA transfection is shown by Adamts4 WB (shown in **c** and **d**), a 0.46 fold expression in the knockdown group against the control group was found. Further, Adamts4 IF (shown in green) also validated the successful knockdown of Adamts4. A decrease in the mean fluorescence intensity from 1 as observed for Scsi treatment set to 0.33 for ATSsi tr group was found. (**e** and **f**). DAPI (shown in blue) was used as nuclear stain. Finally, from qPCR Adamts4 mRNA levels were downregulated from onefold observed for Scsi tr set to 0.25-fold for ATSsi tr group (**g**). n = 3, data analyzed and expressed as mean ± SD. Differences were considered statistically significant for p < 0.05.

Periostin expression is detected by IF staining with anti-Periostin antibody and shows upregulation of the same following H_2_O_2_ and Hyp treatments group in comparison to the control set but this elevation is significantly reduced in ALKI + H_2_O_2_ and ALKI + Hyp groups respectively in comparison to the control group (Fig. 6a, b). Adamts4 loss of function mediated by Adamts4 siRNA transfection (ATSsi Tr) was validated by WB (Fig. 6c, d), IF (Fig. 6e, f) and qPCR (Fig. 6g), all of which validated successful knockdown of Adamts4 compared to the scrambled siRNA (Scsi Tr) treated group. Overall, these data suggest that Periostin activity may be Adamts4 and Tgf-β1 dependent.

### Adamts4 siRNA mediated gene knockdown before H_2_O_2_ and Hypoxia treatment inhibits Adamts4 but does not affect Tgf-β1 expression

**Figure 7:**
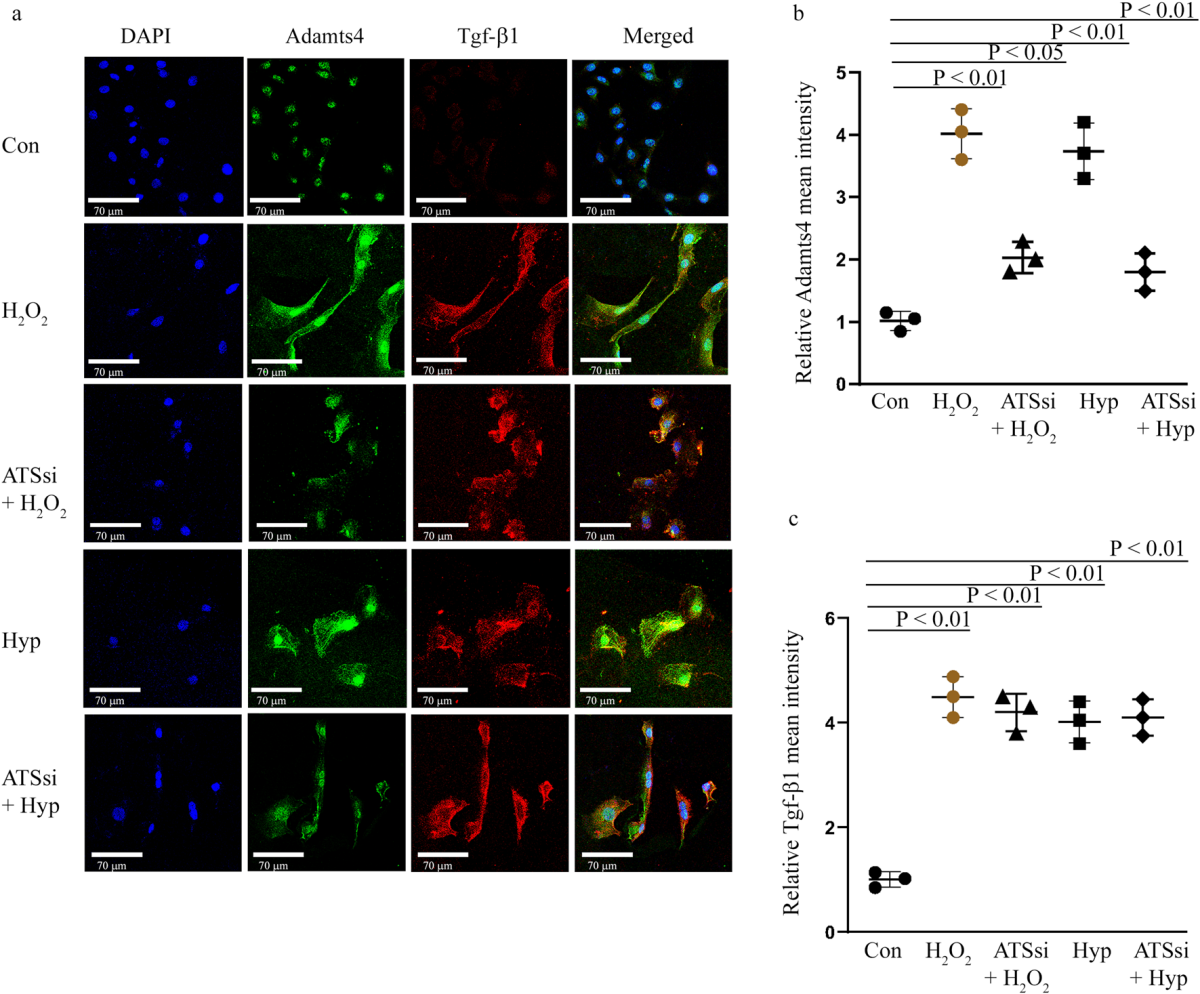
Tgf-β1 expression remains unaffected by Adamts4 knockdown. IF with anti-Adamts4 (shown in green) showed downregulation of Adamts4 following Adamts4 loss of function along with injury treatments. Adamts4 levels were found to decrease to 2 and 1.8 folds in the ATSsi + H_2_O_2_ and ATSsi + Hyp groups in comparison to the 4 and 3.7 folds increase observed for only H_2_O_2_ and Hyp treatments (**a** and **b**) but Tgf-β1 remained largely unaffected by this loss of function of Adamts4 and as shown by staining with anti-Tgf-β1 (shown in red) where the levels of Tgf-β1 were 4.5 and 4.0 for H_2_O_2_ and Hyp groups and 4.2 and 4.1 for ATSsi + H_2_O_2_ and ATSsi + Hyp groups (a and c). DAPI (shown in blue) was used as nuclear stain. Differences between groups H_2_O_2_ and ATSsi + H_2_O_2_, and Hyp and ATSsi + Hyp were not found to be significant. n = 3, data analyzed and expressed as mean ± SD. Differences were considered statistically significant for p < 0.05.

Now to better understand the regulatory hierarchy and interaction between Tgf-β signaling and Adamts4; Adamts4 knockdown experiments are performed. As expected, Adamts4 siRNA transfection before H_2_O_2_ (ATSsi + H_2_O_2_) and hypoxia (ATSsi + Hyp) treatment show a significant reduction in the levels of Adamts4 expression as shown by Adamts4 IF staining (Fig. 7a, b) as compared to the treatment groups; H_2_O_2_ and Hyp, but interestingly Tgf-β1 expression (Fig. 7a, c) remains mostly unaffected by Adamts4 loss of function as no significant difference between H_2_O_2_ and ATSsi + H_2_O_2_ and Hyp and ATSsi + Hyp was found. This finding, therefore, is suggestive of a possible Tgf-β1 function upstream of Adamts4 at least in the context of H_2_O_2_ and hypoxia induced pathological remodeling in cultured H9c2 cells.

### Adamts4 knockdown results in inhibition of Collagen-III and α-SMA expression

**Figure 8:**
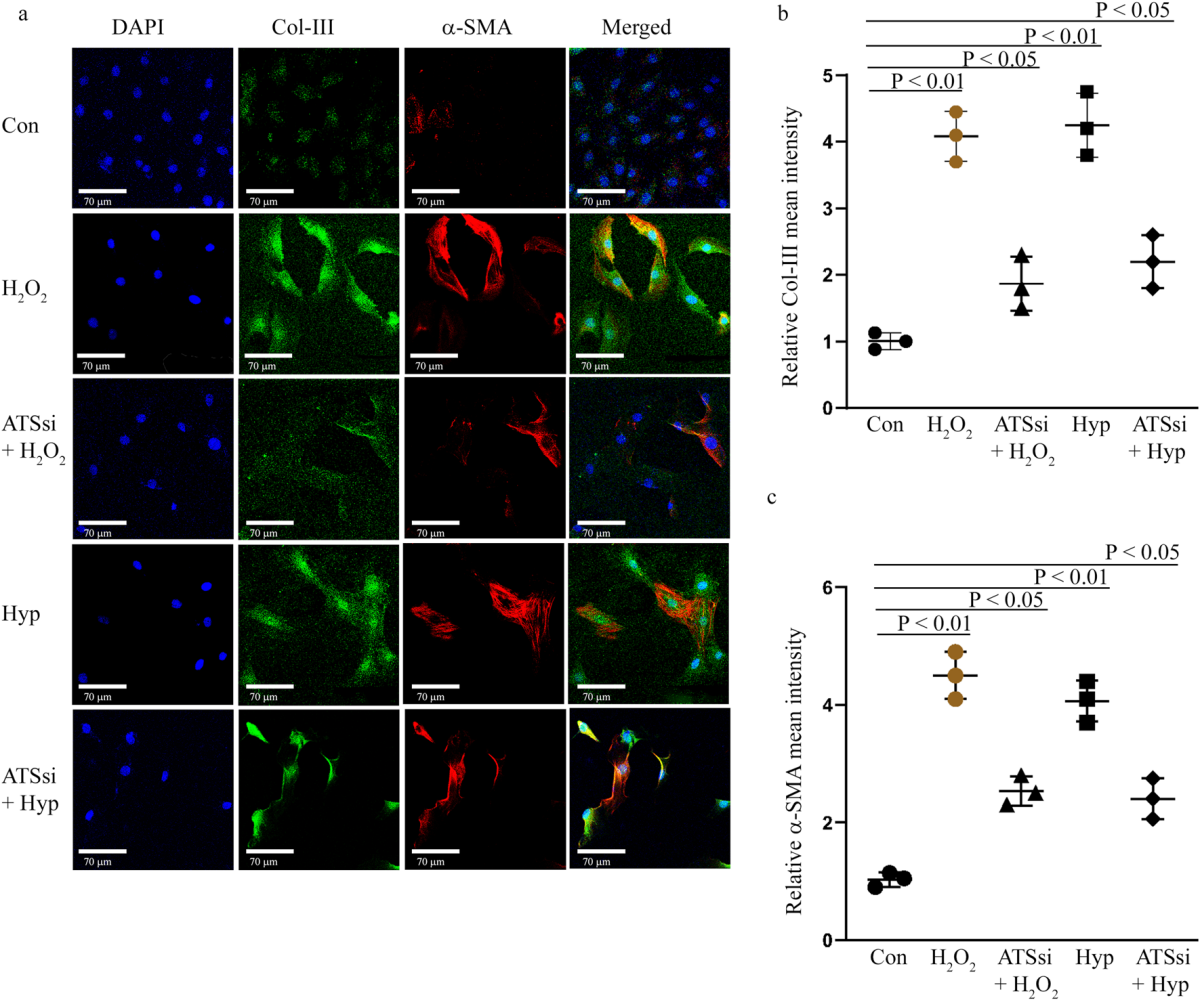
Inhibited expression of Col-III and α-SMA following loss of function of Adamts4. IF with anti-Col-III (shown in green) and anti-α-SMA (shown in red) antibodies show inhibited expression of these markers under ATSsi + H_2_O_2_ and ATSsi + Hyp conditions when compared with H_2_O_2_ and Hyp treated cells (a) Col-III showed a reduction from 4 and 4.25 fold observed for the H_2_O_2_ and Hyp treatment groups in comparison with the control group to 1.8 and 2.2 fold observed in ATSsi + H_2_O_2_ and ATSsi + Hyp groups (**a** and **b**). Similarly, for α-SMA, H_2_O_2_ and Hyp treated cells show a significant 4.5 and fourfold increased expression when compared to control, the ATSsi + H_2_O_2_ and ATSsi + Hyp treated cells show a fold change of 2.5 and 2.4 increments when compared to the control group (**a** and **c**). DAPI (shown in blue) was used as nuclear stain. n = 3, p < 0.05 is considered as significant for differences among groups. Data analysed and expressed as mean ± SD.

Further, markers for injury induced fibrosis or pathological remodeling are determined in Adamts4 dependent manner. Likewise, Adamts4 siRNA mediated knockdown also shows a significant reduction in the expression of Collagen-III and α-SMA in groups where Adamts4 knockdown was performed prior to H_2_O_2_ and hypoxia induction as compared to groups where only injury induction (H_2_O_2_ and hypoxia) was done, shown by Collagen-III and α-SMA IF staining (Fig. 8a–c). Overall, these findings are indicative of an Adamts4 dependent activity under pathological stress conditions induced in H9c2 cells.

### Adamts4 knockdown results in reduced expression of Periostin

**Figure 9:**
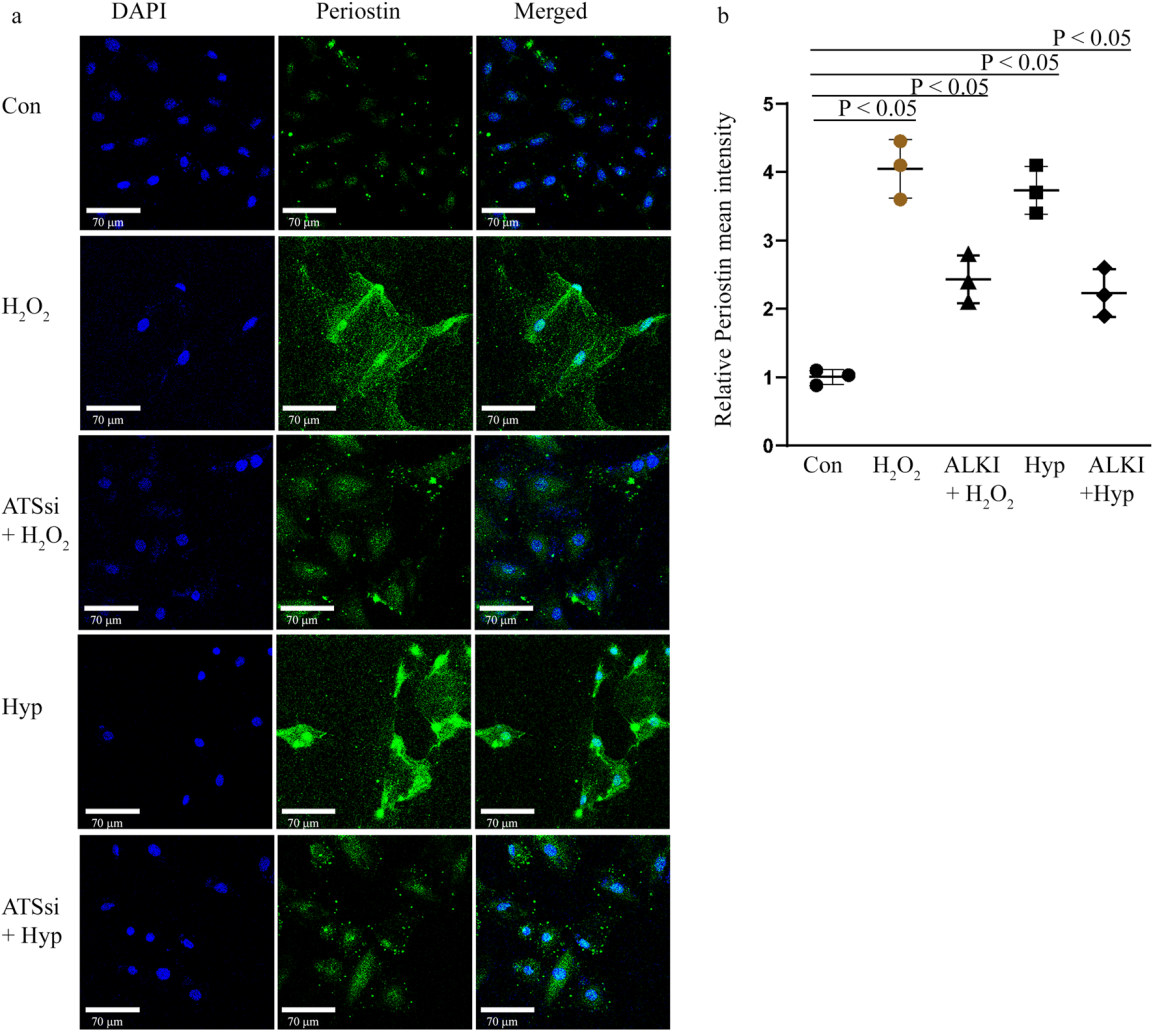
Reduced expression of Periostin following Adamts4 loss of function. IF with anti-Periostin antibody (shown in green colour) shows a fold change of 4 and 3.7 increase in the H_2_O_2_ and Hyp treated group in comparison to the control set, this elevation is significantly reduced 2.4 and 2.2 in ATSsi + H_2_O_2_ and ATSsi + Hyp groups following siRNA mediated knockdown of Adamts4 (**a**). DAPI (shown in blue) was used as nuclear stain. Its quantification is depicted in the graph (**b**). n = 3, data analysed and expressed as mean ± SD. Differences were considered statistically significant for p < 0.05.

Moving along on the same lines as with ALKI treatment, next Periostin expression is detected by IF staining with anti-Periostin antibody post Adamts4 knockdown and this shows upregulated expression of Periostin in H_2_O_2_ and Hyp treated groups in comparison to the control set, this elevation is significantly reduced in ATSsi + H_2_O_2_ and ATSsi + Hyp groups (Fig. 9a, b) showing a significant reduction from only H_2_O_2_ and Hyp treated H9c2 cells. These findings are indicative that Periostin expression may be under Adamts4 regulation.

### The induced expression of Adamts4 and α-SMA in humans with cardiac anomalies

**Figure 10:**
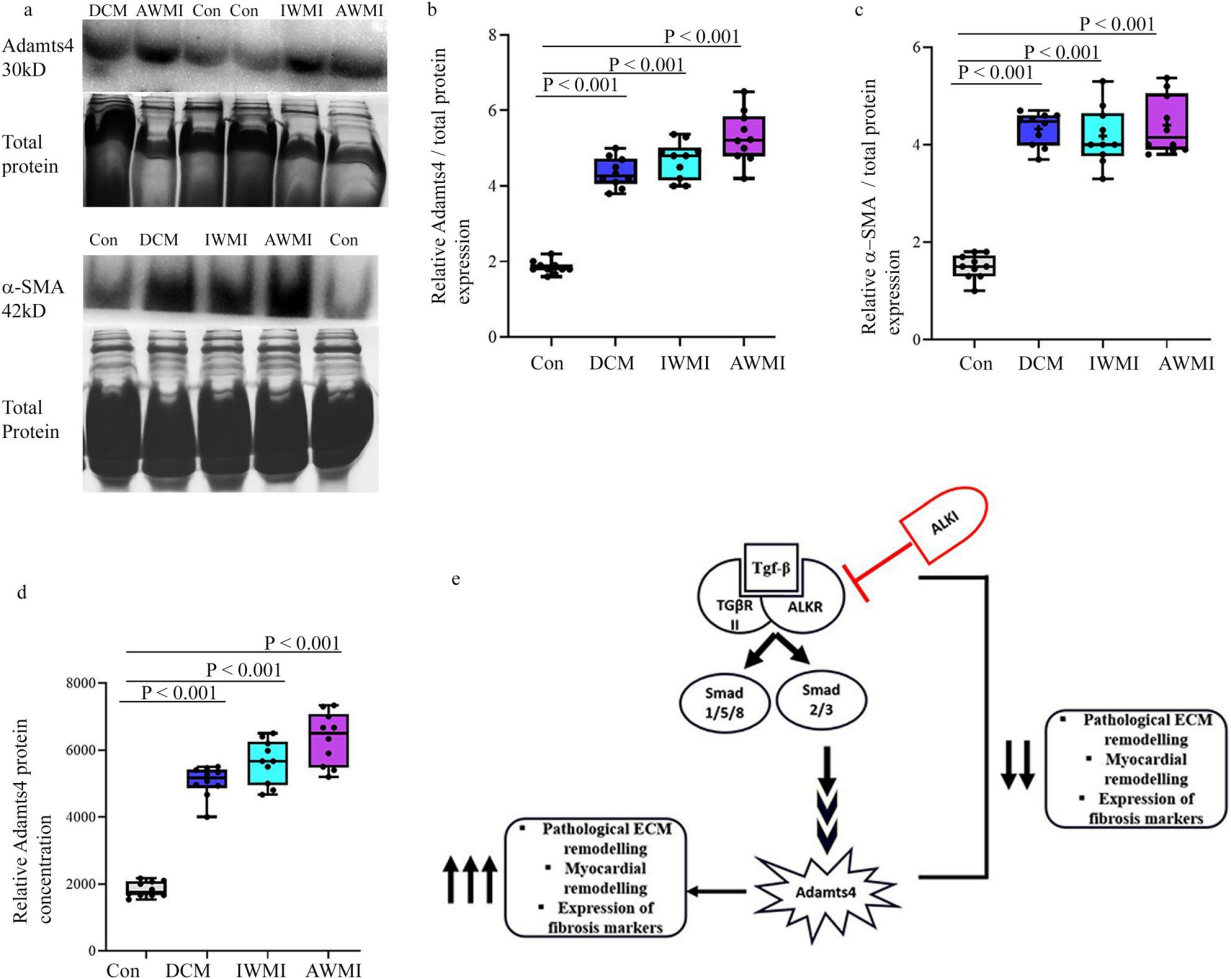
Upregulation of Adamts4 and α-SMA proteins in adult cardiac patients. Adamts4 and α-SMA WB data of affected cardiac patients and control (**a**), its quantification (**b** and **c**). An elevated expression of Adamts4 varying from 3 to 4.5 folds for patients with DCM, 4 to 5.3 folds for IWMI patients and 4 to 6.5 for patients with AWMI and varying in comparison to the control group (with no indicated cardiac abnormalities), where the expression fold varies from 1.6 to 2.3 fold. (**a** and **b**) was observed. The same was true for α-SMA which showed increased fold change varied from 3.7 to 4.7, 3.3 to 5.3, 3.9 to 5.4 for DCM, IWMI, and AWMI groups in comparison with the control set where fold levels for α-SMA varied from 1 to 1.8. (**a** and **c**). Total protein was used as loading control. Further, Adamts4 specific ELISA showed concentration gradient varying from 4666 to 6500 (pg/ml), 5200 to 7333 (pg/ml), and 4000 to 5500 (pg/ml) for DCM, IWMI, and AWMI groups respectively as compared to the concentration gradient observed for the control group which varied from 1533 to 2170 (pg/ml) (**d**). (n = 5 for WB for each group, N = 10 and n = 2 for ELISA for each study group). Data analyzed and expressed as median, 1st and 3rd quartile and range. p < 0.05 was considered a significant difference. The proposed model (**e**) shows the hypothetical hierarchy and inter-relationship between Tgf-β1 and Adamts4 based on our findings. ALKI acting as an inhibitor of Tgf-β1 inhibits the stimulation of Adamts4 by Tgf-β1 which normally is activated under stress or injury conditions and activates downstream molecule Adamts4 and Adamts4 then proceeds with pathological ECM remodeling to restore the damaged physiology of the cells and tissues.

To better correlate our in vivo murine animal model and in vitro findings, in humans, we have used human patients' serum samples with indicated cardiac anomalies. Adamts4 and α-SMA protein levels were assessed by western blot. Both Adamts4 and α-SMA proteins show significantly enhanced expression in patients who have suffered DCM, MI, either AWMI (anterior wall MI) or inferior wall MI (IWMI) (Fig. 10a–c) Furthermore, Adamts4 specific ELISA also validates the same findings (Fig. 10d). Patients with DCM, IWMI or AWMI show significant expression of Adamts4 as compared to the control group. This confirms that Adamts4 is also induced in humans who have a history of cardiac diseases like DCM or have suffered MI. Figure 10e shows a proposed model based on our findings for the interaction and inter-relationship between Adamts4 and Tgf-β1 in post cardiac injury conditions; when the Tgf-β1 expression is inhibited by blocking the binding of Tgf-β1 to one of its two binding receptors (ALK receptors, more specifically ALK 4 and 5), Adamts4 expression is inhibited since it is regulated downstream of Tgf-β1. So, the elevated fibrosis like conditions that escalated following pathological stress induction is inhibited and eventually fibrosis related markers (Col-III, α-SMA and periostin) along with Adamts4 are downregulated following inhibition of Adamts4.

## Discussion

The salient finding of this study is that Adamts4 is upregulated in injury to cardiac muscle. This finding is consistent with previous studies where Adamts4 expression was shown to be enhanced in atherosclerotic plaques^13^ and patients with acute coronary syndrome^15^. Importantly, Adamts4 being a secretory protein in nature, it resides both intracellularly and is also secreted from cardiomyocytes out into the ECM where its function is to regulate the turnover of other proteins by directly affecting other resident cells including but not limited to cardiac fibroblasts, endothelial and smooth muscle cells in the same ECM milieu. However, injury induced function of Adamts4 in other adult cardiac resident cell types is unknown. The expression of Adamts4 significantly decreased from being widespread in the chamber myocardium of developing hearts to very restricted expression in normal adult hearts (Fig. 1) which could imply that its roles in developing and adult hearts are different but the reactivation of Adamts4 following cardiac injury (MI) (Fig. 2) implied that it could be another marker for adult cardiac injury. Since, this expression co-localized with cardiomyocytes, manipulation of Adamts4 was easier to experiment with within H9c2, a cardiomyocyte cell line. Injury inductions through H_2_O_2_ and hypoxia treatment showed upregulation of Adamts4 along with a couple of other fibrosis-related markers like Tgf-β1, Collagen-III, α-SMA, and Periostin which is notably known as a marker for detecting fibroblast to myofibroblast switch^36^ were found to be elevated (Figs. 3 and 6) implying that an injury mediated ECM remodeling could be a cause for the elevation of these markers. Further pre-treatment with SB431542, an inhibitor of ALK4 and 5 (one of the two binding receptors of Tgf-β1) receptor that eventually leads to inhibition of Tgf-β1 also showed inhibition of Adamts4, this inhibition further extended to the expression of Collagen-III, α-SMA and Periostin proteins (Figs. 4, 5 and 6). To better understand the hierarchy supremacy between Adamts4 and Tgf-β1, Adamts4 loss of function study was mediated by Adamts4 siRNA transfection. In groups where Adamts4 knockdown was performed before stress induction, Tgf-β1 expression remained quite unaffected whereas the expression of the other 3 mentioned markers-Collagen-III, α-SMA, and Periostin along with Adamts4 was found to reduce to somewhat similar levels when ALKI pre-treatment with stress induction was done (Figs. 7, 8 and 9). These findings indicated that Adamts4 expression is mediated by Tgf-β1 and also that other ECM and fibrosis related markers including Col-III, α-SMA, and Periostin seem to be regulated by Adamts4 since loss of function of Adamts4 significantly inhibited the expression of these markers following injury to H9c2 cells. Finally, not only our marker of interest, Adamts4 but also α-SMA showed significantly enhanced expression in patients with MI and DCM injury (Fig. 10) indicating a cardiac fibrosis like condition following adult cardiac injury. Although not done here but our future studies are focussed on validating the direct interaction of Adamts4 with Col-III α-SMA, and Periostin through binding/ChIP assays.

The ECM is an integral part of the myocardium. The dynamic composition of cardiac ECM consisting of both structural and non-structural components plays a critical role in cellular events and during pathogenicity^37,38^. Under stress conditions such as Ischemia or MI, the chamber myocardium undergoes intensive ECM remodeling which remains relatively inconspicuous in healthy individuals. Cardiac Fibrosis is often viewed as an expansion of ECM remodelling^39^. Following MI or I/R, there is an acute inflammatory response that suffices for the overexpression of pro-inflammatory cytokines like Tgf-β and interleukins. Tgf-β1 most notably does so by the canonical Smad 2/3 signaling cascade which leads to fibrosis^16,17,39^. This, in turn, activates and synthesizes matrix macromolecule like Adamts^40,41^ family among others as a part of the pro-inflammatory mediated cell signaling cascade and these MMPs then take over the centre stage post-cardiac injury such as MI which leaves behind a pool of necrotic myocytes, it is then that the MMPs like Adamts4 takeover to regulate a turn-over in the synthesis of matrix macromolecules like Collagen-I/III, α-SMA, and Periostin, Tenascin-C to synthesize more matrix macromolecules and fibroblasts which is required to fill in the scar left by necrosis of myocardial cells in order to maintain the physiology of the myocardium since adult cardiomyocytes have very limited proliferation capacity. However, prolonged expansion of ECM could lead to extensive fibrosis and as a resulting stiffness of the myocardium following which ventricular dysfunction could occur which itself may turn fatal and thus inhibition of MMPs like Adamts4 could be one of the possible targets to reduce fibrosis post-cardiac injury^42^ like the one proposed in our model (Fig. 10e) by inhibiting Tgf-β1.

To conclude, Adamts4 is upregulated in response to cardiac stress in *in-vivo*, *in-vitro* and human studies, and this upregulation is mediated by Tgf-β1. This elevation of Adamts4 leads to fibrosis induction as markers related to fibrosis including Collagen-III, α-SMA, and Periostin along with Tgf-β1 is also found to be upregulated but when Adamts4 loss of function is performed, these markers were also found to be inhibited following injury except for Tgf-β1 which remains unaffected indicating that it is upstream of Adamts4 in the signaling cascade. Our findings suggest that Adamts4 after being activated by Tgf-β1 under stress inducing conditions, mediates ECM remodeling and thereafter fibrosis through the functioning of Periostin and α-SMA as determined by our findings. It is possible that Periostin, another secretory ECM molecule known to either regulate or work in tandem with other MMPs^43,44^, works in synchronisation with Adamts4 to regulate ECM remodeling and induce fibrosis. Our findings show an Adamts4 dependent Periostin functioning (depicted by Adamts4 loss of function assay) but to state whether Adamts4 and Periostin directly interact, requires further experimentation to establish any direct association between these two ECM molecules. α-SMA has been known to induce fibroblast contractility and is highly expressed in infarct myofibroblasts to prevent extreme remodeling and thereafter cardiac rupture^45,46^. As our data shows the hallmark expression of Adamts4 in cardiac stress conditions, it can be considered as a novel biomarker for cardiac related injuries leading way for therapeutics to manipulate its expression following cardiac injury for improvement of cardiac functioning. Finally, our work hypothesises that Adamts4 being an ECM secretory protein, after being secreted from chamber myocardium regulates ECM and its remodeling, the details of this mechanism require further depth, understanding and experimentations.

## Methods

### Animal study and experimentation

All experiments involving animals were carried out with the experimental protocols and procedures reviewed and approved by the Cincinnati Children’s Hospital Medical Center Biohazard Safety Committee and Institutional Animal Care and Use Committee. The study was performed in accordance with ARRIVE guidelines and all animal experiments were conducted in compliance with the relevant guidelines. Timed-matings were established, with the morning of an observed copulation plug set at E0.5. For embryonic studies, whole embryos or hearts were harvested on E10.5, E12.5, E14.5, and E18.5 days and proceeded with IHC. For MI induction, 8–10 weeks old adult male Swiss albino mice were used. MI was performed as described previously^24^.

### Immunohistochemistry

Whole embryos and embryonic or adult hearts were harvested, washed in 1X PBS, fixed in 4% paraformaldehyde, dehydrated, and embedded in paraffin. Later the tissue sectioning (5–7 μm) was done using a microtome (Leica Biosystems). For immunohistochemistry, the tissue sections were deparaffinized and hydrated after which antigen retrieval was done using citrate buffer (pH 6) washed in 1X PBS and thereafter, blocking solution (2% BSA with 0.1% Tween20) was added to the sections for 1 h. Later after blocking, the sections were incubated overnight at 4 °C. The following day, the incubated sections were washed in PBS and incubated with secondary antibodies against their respective primary antibodies. The sections were mounted using Vectashied (Vector Labs). The primary antibodies used were Adamts4 (PA1-1749A, Invitrogen), MF20 (1:200, DSHB), and respective secondary antibodies [anti-rabbit/mouse Alexa 488 (green; ab150077, Abcam) or 594 (red; ab150116, Abcam) fluorescent-conjugated; Molecular Probes, 1:100). Topro3 (Molecular Probes, 1:1000) was used as a nuclear stain. Fluorescent images were acquired using a Zeiss LSM 510 confocal microscope and LSM version 3.2 SP2 software.

### Human studies

A total of 30 affected clinical samples were used for this study. Venous blood samples were used for the study. The approval for the collection was ethically approved by the Institutional ethics committee of IPGME&R, Kolkata (IPGME&R/IEC/2019/517). All experiments were approved and performed according to the institutional guidelines of IPGME&R, Kolkata. Informed consent was obtained from all participants. Two broad categories of cardiac conditions were selected namely, Dilated cardiomyopathy (DCM), Myocardial Infarction (including AWMI-anterior wall MI and IWMI-inferior wall MI). MI patients routinely given Statin, high intensity, usually 80 mg of Atorvastatin. Loading doses of Aspirin 300 mg and Clopidogrel 300 mg along with Angiotensin Receptor Blocker (ARB) or Angiotensin Converting Enzyme Inhibitor (ACEI), β-Blocker, nitroglycerin, tranquilizer. DCM cases were given ACEI/ARB/Angiotensin Receptor Neprilysin Inhibitor (ARNI), SGLT2 inhibitors, β-blocker, diuretic, Mineralocorticoid Receptor Antagonist (MRA) and supportive therapy like O_2_. Blood collection timing varied between 12 and 36 h of onset or hospitalisation. Control group consisted of blood collected from healthy volunteers who were not known to be diagnosed with any cardiac ailments or any other lifestyle disease for the record. The characteristics of the patient samples are provided in the following Table 1. Blood samples were obtained in clot vials. The serum was separated by following standard procedure by subjecting the clot vials to centrifugation at 2500 rpm for 10 min at 25 °C. To the isolated serum, protease inhibitor (Genetix GX-2811AR) and phosphatase inhibitor (Genetix GX-0211AR) were added according to the manufacturer’s instructions and stored at − 20 °C until further use for Western Blotting and ELISA.

**Table 1:** Characteristics of clinical sample groups.

Groups	Control (*n* = 10)	DCM ( *n* = 10)	AWMI (*n* = 10)	IWMI (10)
Age	20–55 years	20–55 years	28–60 years	30–60 years
Mean age	44 ± 2 years	43 ± 2 years	47 ± 2 years	47 ± 2 years
Sex, M/F	6/4	6/4	7/3	7/3
Smoker, M/F	0/0	3/0	4/0	1/1

### Cell culture studies

All the *in-vitro* experiments were performed on H9c2, rat ventricular cardiomyoblast cell line. The cells were cultured in Dulbecco’s modified Eagle’s medium (AT007, Himedia) supplemented with 10% FBS (RM10409, Himedia) and 1% penicillin/streptomycin (Pen-Strep) cocktail (15140122, Invitrogen) maintained at a sterile humidified CO_2_ incubator at 5% levels and 37 °C^23^. The cells were used for further experiments at about 75–80% confluency. For experimental treatments, serum-free DMEM was used. For ROS generation, the cells were treated with H_2_O_2_ (100 μM) for 1 h^24,25^. For hypoxia induction, the cells were put in an anaerobic chamber with an anerogas pack (LE200A, Himedia) and anaero indicator tablet (LE065, Himedia) according to the manufacturer’s instructions. The anaerobic chamber with cells was incubated in the CO_2_ incubator for 12 h, the indicator color change confirmed hypoxia induction in the chamber^47^.

### TGF-β inhibitor SB431542 and siRNA treatment

For TGF-β inhibition, the cells were pre-treated with a potent ALK inhibitor, SB431542 (10 μM) (ab120163, Abcam) for 30 min following which the cells were exposed to H_2_O_2_ and hypoxia induction as earlier mentioned^20^. For knockdown of Adamts4, cells were transfected with Adamts4 siRNA (50 pmol) (4390771, Ambion) and Lipofectamine RNAiMax reagent (13778-075, Invitrogen) when the cells were at least 70% confluent for 48 h following manufacturer’s protocol after overnight serum starvation. For negative control, scrambled siRNA (silence select negative control no 1 siRNA) (4390843, Ambion) was used similarly. After 48 h of Adamts4 siRNA treatment, the cells were subjected to H_2_O_2_ and hypoxia treatment as previously described.

### RNA isolation, RT-PCR, and real-time PCR

Total RNA was isolated from control and treated H9c2 cells with Trizol (15596026, Ambion) following the Trizol-Chloroform method. Taking 1 μg of total RNA isolated, cDNA was synthesized using Biorad cDNA synthesis kit (iScript™ Reverse Transcription Supermix for RT, 170-884) in 20 μl of total volume according to manufacturer’s supplied protocol. The cDNA prepared was used for primer optimization and standardization using DNA Taq polymerase (by RT-PCR in reverse time for real-time PCR against primers (BIOTAQ DNA polymerase BIO-21040, Bioline,). The PCRs were performed for 35 cycles using 20 pmol of the rodent primer pairs: Adamts4 (F): 5′-TCATGAACTGGGCCATGTCT-3′ and (R): 5′-GTCAGTGATGAATCGGGGCAC-3′; Hif-1α (F): 5′-CCAGCAGACCCAGTTACAGA-3′ and (R): 5′-TTCCTGCTCTGTCTGGTGAG-3′; β-actin (F): 5′-TCTTCCAGCCCTTCCTTCCTG-3′ and (R): 5′-CACACAGAGTACTTGCGCTC-3′ and Catalase (F): 5′-CCTCGTTCAGGATGTGGTTT-3′ and (R): 5′-TCTGGTGATATCGTGGGTGA-3′, Tgf-β1 (F): 5′-CTGAACCAAGGAGACGGAATAC-3′ and (R): 5′-CTCTGTGGAGCTGAAGCAATAG-3′ and Collagen-III (F): 5′-CTGGTCCTGTTGGTCCATCT-3′ and (R): 5′-ACCTTTGTCACCTCGTGGAC-3′. These primer pairs were further used for real-time PCR studies. Real-time PCR was performed using Bio-Rad real-time PCR kit (172-52 03AP, SSO fast Eva green super mix). The gene expression of the mentioned genes was normalized with β-actin.

### Protein isolation and western blotting

Protein isolation was done using ice-cold mammalian lysis buffer (250 mM NaCl, 50 mM Tris pH 7.5, 0.1% SDS, 1% TritonX, and 5 Mm EDTA) containing protease inhibitor cocktail (Genetix GX-2811AR) and phosphatase inhibitor (Genetix GX-0211AR). The proteins were stored in small aliquots at − 80 °C until further use for Western Blotting (WB). Western blotting was performed as previously described^48^. The immunoblots developed using Clarity™ Western ECL substrate (1705060, Bio-Rad) and scanned using ChemiDocMP (Bio-Rad) as previously described^49^. The antibodies used were Adamts4 (PA1-1749A, Invitrogen) used at a concentration of 2 μg/ml, α-SMA (14-976082, Invitrogen) used at a dilution of 1:500, Tgf-β1 (ab64715, Abcam) used at a concentration of 1 μg/ml and Vimentin (ab17321, Abcam) at a dilution of 1:1000. After overnight incubation at 4 °C with these primary antibodies, the corresponding secondary antibodies-HRP conjugated secondary goat anti-rabbit (ab97051, Abcam) and HRP conjugated goat anti-mouse secondary antibodies (ab97023, Abcam) were added against their respective primary antibodies at a dilution of 1:3000. The SDS gels were also stained with 2.5% coomassie (Brilliant blue G, SRL) and destained with coomassie de-stainer to obtain total protein intensities. The coomassie stained gels were scanned using ChemiDoc MP mentioned earlier. Quantification of intensities was done using ImageJ software (NIH). Figures for unprocessed blots and gels are also included in Supplementary Figs. S5, S6, S7 and S8 with highlighted regions (in red box) corresponding to the data depicted in the main manuscript figures. Full-length blots could not be submitted since the blots were trimmed (according to molecular size of the proteins) before hybridisation with respective antibodies to save on the antibodies and reagents.

### Immunostaining

Immunostaining was performed in H9c2 cells following the protocol previously described^25^ except for the omission of permeabilization steps for staining with antibodies. Adamts4 (PA1-1749A, Invitrogen) used as mentioned earlier, Tgf-β1 (ab 64715, Abcam) used at a concentration of 2 μg/ml, α-SMA (14-976082, Invitrogen) used at 1:400 dilution, collagen III (ab 7778, Abcam) at a dilution of 1:1000 and Periostin (ab14041, Abcam) used at a dilution of 1:500. After overnight incubation at 4 °C with these primary antibodies, the corresponding secondary antibodies Alexa flour 488 goat anti-rabbit secondary antibody (ab150077, Abcam) and Alexa flour 594 goat anti-mouse secondary antibody (ab150116, Abcam) were added against their respective primary antibodies. All cell nuclei were stained with DAPI (D9542, Sigma). Images were acquired using Leica confocal microscope and LasX software. The fluorescence intensities were measured and quantified with the help of ImageJ software (NIH).

### Enzyme linked immunosorbent assay (ELISA)

ELISA kit (ab213753, Abcam) was used to perform ELISA. Serum proteins were diluted with sample buffer in the ratio of 1:10 and samples were loaded onto the wells in duplicates. ELISA was performed as stated by the manufacturer’s protocol.

### Statistical analysis

All the results are mostly represented as mean ± Standard Deviation of mean (SD) or as median, quartiles and range (Fig. 10). Statistical analyses were done using student’s unpaired two-tailed T-test and one-way ANOVA for more than 2 groups. GraphPad Prism 9.3.1 was used for statistical analysis. Differences among the groups were considered statistically significant for *P* < 0.05.

## Supplementary Information

Supplementary Information.

## Data Availability

All the generated data used to support the findings of this study are either included within the article or in the supplementary file.
