# Supplementary material for: Identification of Adamts4 as a novel adult cardiac injury biomarker with therapeutic implications in patients with cardiac injuries
Source: Sci Rep. 2022 Jun 14;12:9898. doi: 10.1038/s41598-022-13918-3 (PMC9197855; doi:10.1038/s41598-022-13918-3)
Supplement: Supplementary file 1 — Supplementary Information. [file 41598_2022_13918_MOESM1_ESM.docx]

**Supplementary Information**

**Identification of Adamts4 as a novel adult cardiac injury biomarker with therapeutic implications in patients with cardiac injuries.**

**Riffat Khanam^1^, Arunima Sengupta^2^, Dipankar Mukhopadhyay^3^, Santanu Chakraborty^1^**

^1^Department of Life Sciences, Presidency University, Kolkata 700073, India

^2^Department of Life science & Biotechnology, Jadavpur University, Kolkata 700032, India

^3^Department of Cardiology, Institute of Post Graduate Medical Education & Research (IPGME&R), SSKM Hospital, Kolkata 700020, India. All correspondence should be addressed to Santanu Chakraborty^1^ (email: [santanu.dbs@presiuniv.ac.in](mailto:santanu.dbs@presiuniv.ac.in))

**Supplementary Results**

**H9c2 cell morphology after ROS and Hypoxia injury induction.** The change in morphology with reduction in size of H9c2 cells subjected to H_2_O_2_ and hypoxia as compared to control group is depicted by Phalloidin immunostaining in Supplementary Fig. S1a. Staining with Alexafluor conjugated Phalloidin validated the reduction in cell size after both injury inductions. A reduction in cell size from 260 μm^2^ for the control group to 180 μm^2^ and 170 μm^2^ for H_2_O_2_ and hypoxia treatment groups respectively (Supplementary Fig. S1a and b).

**Vimentin over expression in murine adult primary cardiac fibroblasts after ROS and Hypoxia injury.** Vimentin, an intermediate filament expressed and associated with fibroblast differentiation to myofibroblast^1,2^ was found to show enhanced expression when the cultured adult murine primary fibroblasts were subjected to ROS and hypoxia injury as compared to control group as shown in panel a. A 3.2 and 2.6 fold increase in fluorescence intensities after immunostaining with anti-Vimentin antibody in cells treated with H_2_O_2_ and hypoxia respectively was found as shown in panel b (Supplementary Fig. S2a and b).

**Upregulation of Periostin and α-SMA in** **murine** **adult primary cardiac fibroblasts after ROS and Hypoxia injury.** Similar to Vimentin, Periostin and α-SMA marker expressions (measured by mean fluorescence intensities) after immunostaining with the anti-Periostin and anti-α-SMA antibodies was found to be significantly upregulated after both injury treatments. A 2.8 and 3 fold upregulated Periostin expression was found in H_2_O_2_ and hypoxia treated groups as compared to control (Supplementary Fig. S3a and b) while a 3.3 and 3.6 fold upregulation in the levels of α-SMA was found for H_2_O_2_ and hypoxia treated groups respectively (Supplementary Fig S3a and c).

**Re-activation of Adamts4 and Tgf-β1 in murine adult primary cardiomyocytes after ROS injury.** The isolation of primary adult mice cardiomyocytes after culture captured under a Differential Interface Contrast (DIC) microscope is shown in Supplementary Fig. S4a. The isolation of primary cardiomyoytes were further validated by anti-MF20 antibody staining (Supplementary Fig. S4b). Immunostaining with anti-Adamts4 and anti-Tgf-β1 showed overexpression of Adamts4 and Tgf-β1 (shown in Supplementary Fig. S4c) after ROS induction. A 3.6 and 3 fold elevation in the levels of Adamts4 and Tgf-β1 respectively (measured as fluorescence intensity) is found after ROS induction as compared to control group (Supplementary Fig. S4d and e). This finding in primary cardiomyocytes is in affirmation with our findings in cultured H9c2, a cardiomyocyte cell line.

**Supplementary Methods**

**Cell culture studies:** All the *in-vitro* experiments were performed on H9c2, rat ventricular cardiomyocyte cell line. The cells were cultured in Dulbecco’s modified Eagle’s medium (AT007, Himedia) supplemented with 10% FBS (RM10409, Himedia) and 1% penicillin /streptomycin (Pen-Strep) cocktail (15140122, Invitrogen) maintained at a sterile humidified CO_2_ incubator at 5% levels and 37°C.^3^ Murine adult primary mice cardiac fibroblasts were isolated and maintained in cell culture as described previously^4^ on collagen coated plates. For primary cardiomyocyte culture, protocol described previously^5^ was followed except for the Langendorff perfusion set-up which was replaced by mechanical perfusion set-up. The cells were used for further experiments at about 75-80% confluency. For experimental treatments, serum-free media was used. For ROS generation, the cells were treated with H_2_O_2_ (100 μM) for 1 hour.^6,7^ For hypoxia induction, the cells were put in an anaerobic chamber with an anerogas pack (LE200A, Himedia) and anaero indicator tablet (LE065, Himedia) according to the manufacturer’s instructions. The anaerobic chamber with cells was incubated in the CO_2_ incubator for 12 hours, the indicator color change confirmed hypoxia induction in the chamber.^8^ Since primary adult cardiomyocytes could not be sustained for long duration only ROS injury was done.

**Immunostaining.** Immunostaining was performed in murine primary adult cardiac fibroblast and cardiomyocyte cells following the protocol previously described^7^ except for the omission of permeabilization steps for staining with antibodies. Phalloidin (8878, CST) used at a concentration of 1:400, Adamts4 (PA1-1749A, Invitrogen) used at a concentration of 2 μg/ml Tgf-β1 (ab 64715, Abcam) used at a concentration of 2 μg/ml, MF20 (DSHB) used at a dilution of 1:200, Vimentin (ab17321, Abcam) used at dilution 1:500, α-SMA (14-976082, Invitrogen) used at 1:400 dilution, Periostin (ab14041, Abcam) used at a dilution of 1:500. After overnight incubation at 4°C with these primary antibodies, the corresponding secondary antibodies Alexa flour 488 goat anti-rabbit secondary antibody (ab150077, Abcam) and Alexa flour 594 goat anti-mouse secondary antibody (ab150116, Abcam) were added against their respective primary antibodies. All cell nuclei were stained with DAPI (D9542, Sigma). All DIC and fluorescence images were acquired using Leica Fluorescence microscope and LasX software. The fluorescence intensities were measured and quantified with the help of ImageJ software (NIH).

**Statistical analysis:** All the results are represented as mean ± Standard deviation of the mean (SD). Statistical analyses were done using student’s unpaired two-tailed T-test and one-way ANOVA for more than 2 groups. Differences among the groups were considered statistically significant for P < 0.05.

**Supplementary References**

1. Chaurasiaa, S. S., Kaura, H., deMedeirosa,F. W., Smith, S. D., Wilson, S. E. Exp Eye Res. 2009 August ; 89(2): 133–139. doi:10.1016/j.exer.2009.02.022.

2.	Hinderer, S. & Schenke-Layland, K. Cardiac fibrosis – A short review of causes and therapeutic strategies. *Adv. Drug Deliv. Rev.* **146**, 77–82 (2019).

3.	Fernández-Rojas, B., Vázquez-Cervantes, G. I., Pedraza-Chaverri, J. & Gutiérrez-Venegas, G. Lipoteichoic acid reduces antioxidant enzymes in H9c2 cells. *Toxicol. Reports* **7**, 101–108 (2020).

4.	Sahadevan, P. & Allen, B. G. Isolation and culture of adult murine cardiac atrial and ventricular fibroblasts and myofibroblasts. *Methods* (2021). doi:10.1016/j.ymeth.2021.04.004

5.	Li, D., Wu, J., Bai, Y., Zhao, X. & Liu, L. Isolation and culture of adult mouse cardiomyocytes for cell signaling and in vitro cardiac hypertrophy. *J. Vis. Exp.* 2–9 (2014). doi:10.3791/51357

6.	Sengupta, A., Molkentin, J. D., Paik, J. H., DePinho, R. A. & Yutzey, K. E. FoxO transcription factors promote cardiomyocyte survival upon induction of oxidative stress. *J. Biol. Chem.* **286**, 7468–7478 (2011).

7.	Sen, P., Polley, A., Sengupta, A. & Chakraborty, S. Tbx20 promotes H9c2 cell survival against oxidative stress and hypoxia in vitro. *Indian Journal of Experimental Biology*. **57**, 643–655 (2019).

8.	Ma, Y. *et al.* DPP-4 inhibitor anagliptin protects against hypoxia-induced cytotoxicity in cardiac H9C2 cells. *Artif. Cells, Nanomedicine Biotechnol.* **47**, 3823–3831 (2019).

**Supplementary Figure Legends**

Supplementary Figure S1: **Cell morphology after ROS and hypoxia injury.** Immunostaining with Alexafluor conjugated Phalloidin showed reduction in cell size from 260 μm^2^ for the control group to 180 μm^2^ and 170 μm^2^ for H_2_O_2_ and hypoxia treatment groups respectively (shown in panel a and b). n=3, data analyzed and expressed as mean ± SD. Differences were considered statistically significant for *p* < 0.05.

Supplementary Figure S2: **Enhanced expression of Vimentin in adult primary cardiac fibroblasts following ROS and hypoxia injury treatment.** Staining with anti-Vimentin antibody (shown in green) showed a 3.2 and 2.6 fold change in the mean fluorescence intensity of Vimentin post H_2_O_2_ and hypoxia treatments as compared to control group (a and b). DAPI (in blue) was used as nuclear stain. n=3, data analyzed and expressed as mean ± SD. Differences were considered statistically significant for *p* < 0.05.

Supplementary Figure S3: **Upregulated expression of Periostin and α-SMA in murine adult primary cardiac fibroblasts following ROS and hypoxia injury treatment.** Immunostaining with anti-Periostin (shown in green) showed a 2.8 and 3 fold elevation in fluorescence intensity of Periostin in H_2_O_2_ and hypoxia treated groups as compared to control (a and b) while a 3.3 and 3.6 fold upregulation (in the fluorescence intensity) in the levels of α-SMA (shown in red) was observed for H_2_O_2_ and hypoxia treated groups respectively (a and c). DAPI (shown in blue) was used as nuclear stain. n=3, data analyzed and expressed as mean ± SD. Differences were considered statistically significant for *p* < 0.05.

Supplementary Figure S4: **Overexpression of Adamts4 and** **Tgf-β1 in murine adult primary cardiomyocytes after ROS injury.** The isolation of adult primary cardiomyocytes depicted through DIC images shows the culture status. Few rod shaped cardiomyocytes were observed (insets in a), rest round cardiomyocytes. Further, the isolation was validated with anti-MF20 (shown in red) antibody staining to mark the cardiomyocytes (b). The expression pattern of Adamts4 (shown in green) and Tgf-β1 (shown in red) after H_2_O_2_ treatment showed a clearly enhanced expression in the fluorescence intensities of both Adamts4 and Tgf-β1 by 3.6 and 3 fold respectively (c, d and e). DAPI (shown in blue) was used as nuclear stain. n=3, data analyzed and expressed as mean ± SD. Differences were considered statistically significant for *p* < 0.05.

Supplementary Figures. S5**: Unprocessed western blot and gel images of data used in MS fig. 3f.** From a to e, western blots of Vimentin, α-SMA (including an additional replicative blot shown in panel c), Adamts4 and Coomassie gel (e) depicting total protein. The red highlighted area shows the region of the blot used as representative data in the main MS.

Note: Full length blots could not be submitted since the blots were trimmed (according to molecular size of the protein) before hybridisation with respective antibodies to save on the antibody(ies).

Supplementary Figures. S6**: Unprocessed western blot and gel images of data used in MS figs. 4d and g.** From a to e, western blots of Adamts4 (including an additional replicative one depicted in panel b), Tgf-β1 and gel images (d and e) of Total protein used in MS Figs. d and g. The red highlighted area shows the region of the blot used as representative data in the main MS.

Note: Full length blots could not be submitted since the blots were trimmed (according to molecular size of the proteins) before hybridisation with respective antibodies to save on the antibody(ies).

Supplementary Figures. S7**: Unprocessed western blot and gel images of data used in MS fig.6c.** Western blot of Adamts4 (shown in panel a) and gel depicting total protein (shown in panel b) as shown in MS Fig. 6c. The region highlighted in red is the area used to represent data in the MS file figure.

Note: Full length blots could not be submitted since the blots were trimmed (according to molecular size of the proteins) before hybridisation with respective antibodies to save on the antibody(ies).

Supplementary Figures. S8**: Unprocessed western blot and gel images of data used in MS fig.10a.** From a to e , western blots of Adamts4 (including an additional replicative one shown in panel c), α-SMA (shown in panel d), gel images depicting total protein ( shown in panel b and e) represented in MS Fig. 10a. The region highlighted in red is the area used t o represent data in the MS file figure.

Note: Full length blots could not be submitted since the blots were trimmed (according to molecular size of the proteins) before hybridisation with respective antibodies to save on the antibody(ies).
